# Regiochemical Analysis of the ProTide Activation Mechanism

**DOI:** 10.1021/acs.biochem.4c00176

**Published:** 2024-07-03

**Authors:** Kyle M. Glockzin, Tamari Narindoshvili, Frank M. Raushel

**Affiliations:** ‡ Department of Chemistry, Texas A&M University, College Station, Texas 77843, United States; § Department of Biochemistry & Biophysics, Texas A&M University, College Station, Texas 77843, United States

## Abstract

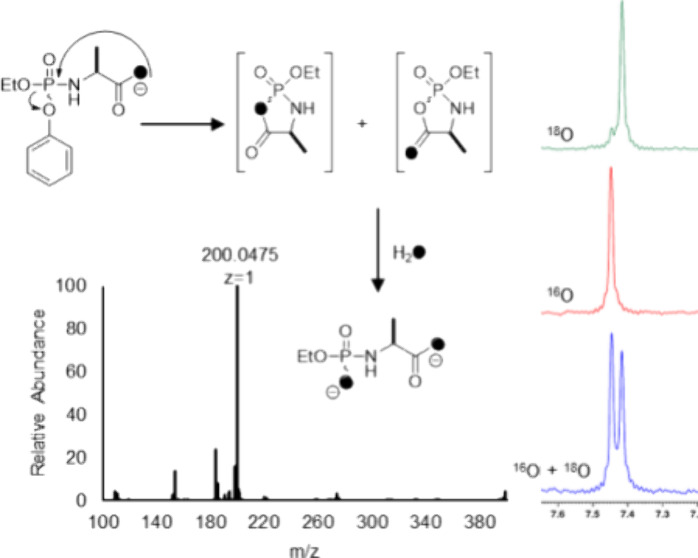

ProTides are nucleotide analogues used for the treatment of specific viral infections. These compounds consist of a masked nucleotide that undergoes *in vivo* enzymatic and spontaneous chemical transformations to generate a free mononucleotide that is ultimately transformed to the pharmaceutically active triphosphorylated drug. The three FDA approved ProTides are composed of a phosphoramidate (P–N) core coupled with a nucleoside analogue, phenol, and an l-alanyl carboxylate ester. The previously proposed mechanism of activation postulates the existence of an unstable 5-membered mixed anhydride cyclic intermediate formed from the direct attack of the carboxylate group of the l-alanyl moiety with expulsion of phenol. The mixed anhydride cyclic intermediate is further postulated to undergo spontaneous hydrolysis to form a linear l-alanyl phosphoramidate product. In the proposed mechanism of activation, the 5-membered mixed anhydride intermediate has been detected previously using mass spectrometry, but the specific site of nucleophilic attack by water (P–O versus C–O) has not been determined. To further interrogate the mechanism for hydrolysis of the putative 5-membered cyclic intermediate formed during ProTide activation, the reaction was conducted in ^18^O-labeled water using a ProTide analogue that could be activated by carboxypeptidase Y. Mass spectrometry and ^31^P NMR spectroscopy were used to demonstrate that the hydrolysis of the mixed anhydride 5-membered intermediate occurs with exclusive attack at the phosphorus center.

## Introduction

**Figure 1:**
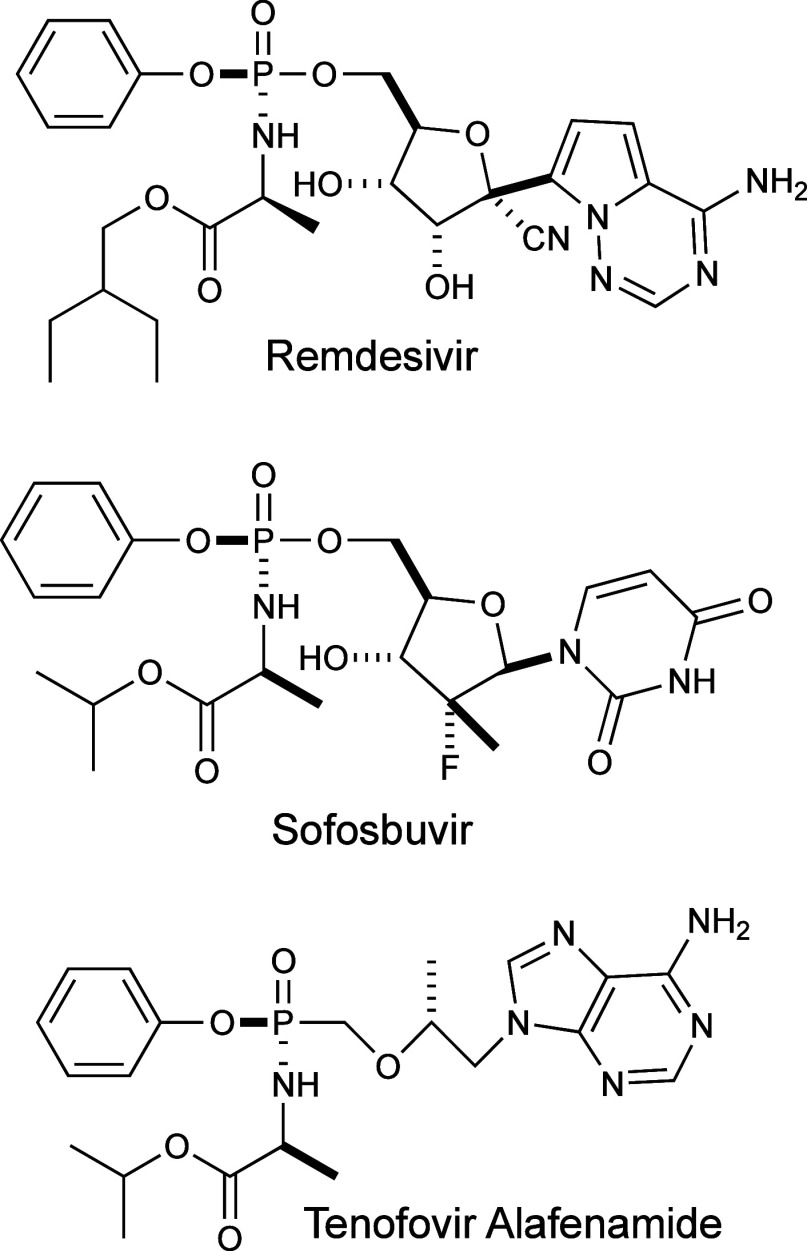
Chemical structures of three currently FDA approved ProTide pharmaceuticals.

Phosphoramidate prodrugs (ProTides) were first engineered and developed by McGuigan and his group at Cardiff University.^1−3^ Originally developed to improve treatment for HIV, ProTides have become a leading therapeutic method for other viral infections.^4,5^ ProTides consist of a phosphoramidate (P–N) core coupled with a nucleoside analogue, a phenol, and an l-alanyl carboxylate ester.^6^ To date, the FDA has approved Sofosbuvir, Tenofovir Alafenamide, and Remdesivir for use as treatments for hepatitis C, HIV, and COVID-19, respectively.^7−9^ The structures of these compounds are highlighted in Figure 1.

**Figure 2:**
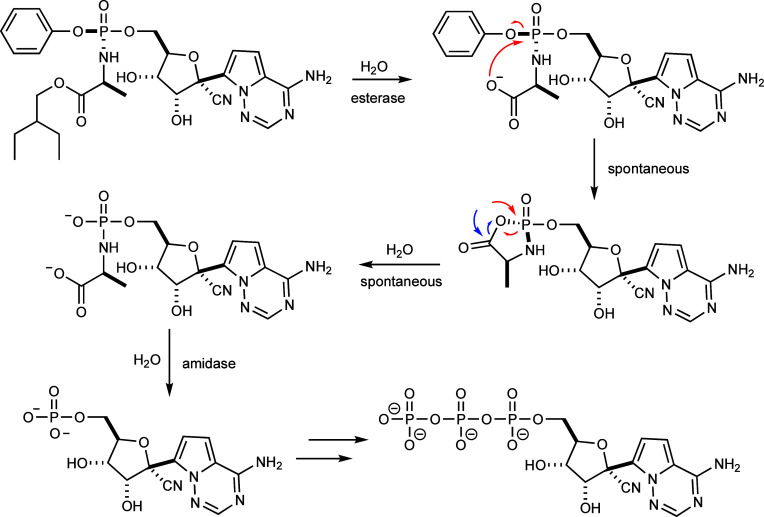
Proposed *in vivo* mechanism of activation for the ProTide Remdesivir.^22,23^ In this mechanism, the cyclic intermediate can potentially be attacked by water at either the phosphate or carboxylate to generate the same product.

The proposed *in vivo* mechanism of activation for ProTides begins with the hydrolysis of the carboxylate ester by an internal peptidase or esterase as illustrated in Figure 2 for the activation of Remdesivir.^10−14^ The next step is hypothesized to be the spontaneous attack of the phosphorus core by the newly formed carboxylate, resulting in the release of phenol and formation of a 5-membered mixed anhydride cyclic intermediate.^6,12^ Recent mass spectrometry experiments have provided support for the 5-membered cyclic intermediate.^15,16^ The mixed anhydride cyclic intermediate has been proposed to be spontaneously attacked by water to form a linear phosphoramidate product.^6,17,18^ The linear phosphoramidate product from the putative cyclic intermediate could result from the attack of water at the phosphate or carboxylate ester. In either case, the product is the same. The l-alanyl moiety is subsequently hydrolyzed to generate the free nucleotide that is then phosphorylated in the cell to provide the nucleotide triphosphate that is the ultimate inhibitor.^19−21^

The site of hydrolysis of the proposed cyclic intermediate has not been determined (P–O vs C–O bond cleavage).^6,15,16^ Here, we have further interrogated the mechanism of ProTide activation using ESI-mass spectrometry and ^31^P NMR spectroscopy when the products are formed in oxygen-18 labeled water. The results from the oxygen-18 labeling conclusively demonstrate that the cyclic intermediate is hydrolyzed via the attack of water at the phosphorus center rather than the carboxylate.

## Materials and Methods

### Materials

General lab supplies were purchased from SigmaAldrich and VWR. *S. cerevisiae* carboxypeptidase Y was purchased from SigmaAldrich and prepared according to the manufacturer’s specifications. ^18^O-labeled water (≥97%) was purchased from Medical Isotopes Inc.

### Synthesis of ProTide Analogue **1a**

A stirred solution of ethyl dichlorophosphate (96%, 0.78 mL, 6.82 mmol, 1.1 equiv) in 20 mL of anhydrous dichloromethane was cooled to −78 °C. Phenol (89%, 0.65 mL, 6.0 mmol, 1 equiv) was added, followed by the dropwise addition of triethylamine (6.0 mmol, 0.84 mL, 1.0 equiv). The reaction mixture was allowed to warm to room temperature and then stirred for 4 h. The reaction mixture was cooled to −78 °C, l-alanine isopropyl ester hydrochloride (1.04 g, 6.2 mmol, 1.0 equiv) was added, and then triethylamine (1.7 mL, 12.0 mmol, 2.0 equiv) was added dropwise. The reaction mixture was allowed to warm to room temperature (23 °C) and stirred for an additional 18 h. The mixture was filtered and concentrated, and the residue was purified by silica gel column chromatography (4:3 and 1:1, hexanes/ethyl acetate mixtures). The final product (compound **1a**) was a colorless oil as a mixture of two diastereomers yielding 0.68 g (35%).

Spectroscopic analysis: ^1^H NMR (400 MHz, CDCl_3_) δ 7.36–7.30 (M, 2H), 7.27–7.21 (m, 2H), 7.19–7.13 (m, 1H), 5.09–4.98 (m, 1H), 4.25–4.10 (m, 2H), 4.05–3.93 (m, 1H), 3.56–3.46 (m, 1H), 1.40–1.32(m, 6H), 1.30–1.21 (m, 6H); ^31^P NMR (160 MHz, CDCl_3_) δ 2.33 (s), 2.21 (s); (ESI^+^) *m*/*z* [M + H]^+^ calculated for C_14_H_23_NO_5_P: 316.1311, found: 316.1308.

### Synthesis of ProTide Analogue **1b**

**Figure 3:**
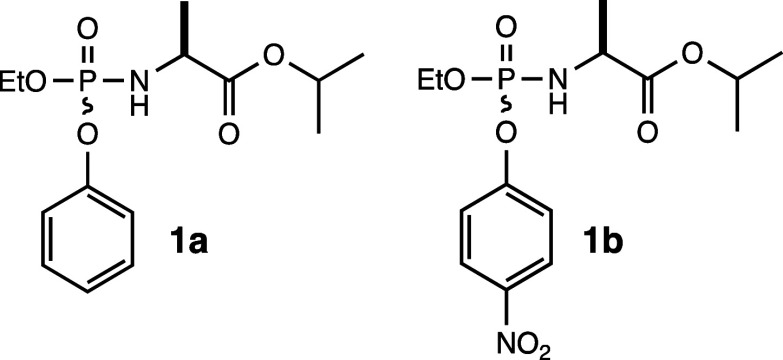
Analogues of ProTides synthesized for this investigation. The diastereomeric mixtures are racemic at the phosphorus center but chiral at the α-carbon of the l-alanine moiety.

l-Alanine isopropyl ester hydrochloride (1.04 g, 6.2 mmol, 1.0 equiv) was suspended in 20 mL of anhydrous dichloromethane and cooled to −78 °C. Ethyl dichlorophosphate (96%, 0.78 mL, 6.82 mmol, 1.1 equiv) was added, followed by the dropwise addition of triethylamine (1.7 mL, 12.4 mmol, 2.0 equiv). The cooling bath was removed, and stirring continued for an additional 3 h at room temperature. The reaction mixture was then cooled to 0 °C, and *p*-nitrophenol (0.77 g, 5.6 mmol, 0.9 equiv) was added, followed by the addition of triethylamine (0.85 mL). The reaction mixture was allowed to stir at room temperature (23 °C) for 18 h. The mixture was filtered, concentrated, and the residue purified by silica gel column chromatography (4:1, 2:1, and 1:1 hexanes/ethyl acetate mixtures). The final product was a colorless oil as a mixture of diastereomers yielding 0.65 g (29%). The structures of compounds **1a** and **1b** are presented in Figure 3.

Spectroscopic analysis: ^1^H NMR (400 MHz, CDCl_3_) δ 8.25 (d, *J* = 8.4 Hz, 2H), 7.44–7.37 (m, 2H), 5.11–4.98 (m, 1H), 4.23–4.18 (m, 2H), 4.05–3.93 (m, 1H), 3.68–3.58 (m, 1H), 1.44–1.35 (m, 6H), 1.31–1.22 (m, 6H); ^31^P NMR (160 MHz, CDCl_3_) δ 2.00 (s), 1.90 (s). (ESI^+^) *m*/*z* [M + H]^+^ calculated for C_14_H_22_N_2_O_7_P: 361.1162, found: 361.1159.

### UV–vis Spectra

Spectral analyses of compounds **1a** and **1b** were performed using a SpectraMax 384 UV–vis spectrophotometer from Molecular Devices. Compounds **1a** and **1b** were resuspended and diluted with dimethylformamide (DMF). The UV–vis spectra of each compound were obtained before and after the addition of carboxypeptidase Y to initiate the hydrolysis of the isopropyl carboxylate ester. Reactions (1.0 mL) contained 50 mM triethanolamine-HCl (pH 8.0) and 1.0 mM of compound **1a** or 100 μM of compound **1b** (1% DMF). Reactions were initiated by the addition of 1.0 μM carboxypeptidase Y at 25 °C.

### Stereoselective Hydrolysis of Phosphoramidates

Carboxypeptidase Y was selected to hydrolyze the isopropyl carboxylate ester based on previous studies and for its ability to hydrolyze amino acid carboxylate esters.^24−26^ Carboxypeptidase Y was dissolved in 50 mM MES (pH 6.75) and aliquots stored at −80 °C. Compounds **1a** and **1b** were dissolved and diluted in DMF. Enzyme assays (1.0 mL) were conducted at 25 °C in quartz cuvettes containing 50 mM triethanolamine-HCl (pH 8.0), 400 μM compound **1a** or 200 μM compound **1b** (1% DMF). Assays were initiated by the addition of enzyme and monitored by UV–vis spectroscopy at 271 (Δε_271_ = 1,320 M^–1^ cm^–1^) or 348 nm (Δε_348_ = 4,840 M^–1^ cm^–1^) for compounds **1a** and **1b**, respectively. Three concentrations of carboxypeptidase Y were used to hydrolyze **1a**, 200, 50, and 10 nM, and for the hydrolysis of **1b**, 100, 25, and 5 nM were used.

The ability of carboxypeptidase Y to differentially hydrolyze the two diastereomers of compounds **1a** and **1b** was tested using ^31^P NMR spectroscopy. Enzyme assays (500 μL) were conducted in 100 mM triethanolamine-HCl (pH 8.0) using either 4.0 mM **1a** or **1b** (2% DMF) at 30 °C. Reactions were initiated by the addition of carboxypeptidase Y and ^31^P NMR spectra were taken periodically.

### Positional Hydrolysis of Putative Cyclic Intermediate

Reactions (100 μL) were conducted in 90% ^18^O-labeled water, 50 mM triethanolamine-HCl (pH 8.0), and either 4.0 mM **1a** or **1b** (2% DMF). Reactions were initiated by the addition of 1.9 μM carboxypeptidase Y and incubated at 25 °C for 1 h. Samples were analyzed by electrospray ionization mass spectrometry (ESI-MS) to determine the extent of incorporation of ^18^O to the ultimate reaction product. Control reactions were conducted in ^16^O-water.

Additional reactions (500 μL) were also conducted in 90% ^18^O-labeled water, 50 mM triethanolamine-HCl (pH 8.0), and 4.0 mM of either compound **1a** or **1b** (2% DMF). Reactions were initiated by the addition of 1.9 μM carboxypeptidase Y and incubated at 25 °C for 1 h. After the reactions were complete, 55 μL of D_2_O was added to the solution and the sample analyzed by ^31^P NMR spectroscopy. Control reactions were carried out in ^16^O-water for both compounds.

### Rate of Cyclization

Reactions (500 μL) were performed in 100 mM triethanolamine-HCl (pH 8.0), 4.0 mM **1a** (2% DMF), and 10% D_2_O at 30 °C. The reaction was initiated by the addition of 3.9 μM carboxypeptidase Y and monitored using ^31^P NMR spectroscopy at 6 min intervals. The rate of product formation and substrate decay were calculated using eqs 1 and 2, respectively. Additionally, the rate of cyclization at 25 °C was determined by UV–vis spectroscopy using 0.40, 0.60, 0.80, and 1.0 μM carboxypeptidase Y in 50 mM triethanolamine-HCl (pH 8.0) starting from 400 μM of compound **1a**. These reactions were monitored at 271 nm, and the data were fit to eq 1.

### Data Analysis

Time courses were processed using SigmaPlot 11.0. Data for the hydrolysis reactions and the determination of the kinetic constants were analyzed as follows. The time courses for the carboxypeptidase Y catalyzed hydrolysis reactions were fit to a single exponential (eq 1) where *k* is the first-order rate constant for the hydrolysis of substrate. For reactions in which the stereoselectivity for enzymatic hydrolysis was biphasic, the entire time course was fit to a double exponential (eq 3), where *k*_1_ is the rate constant for hydrolysis of the first diastereomer and *k*_2_ is the rate constant for hydrolysis of the slower diastereomer.
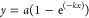
1
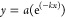
2
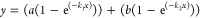
3

## Results and Discussion

### Synthesis of ProTide Analogues

**Figure 4:**
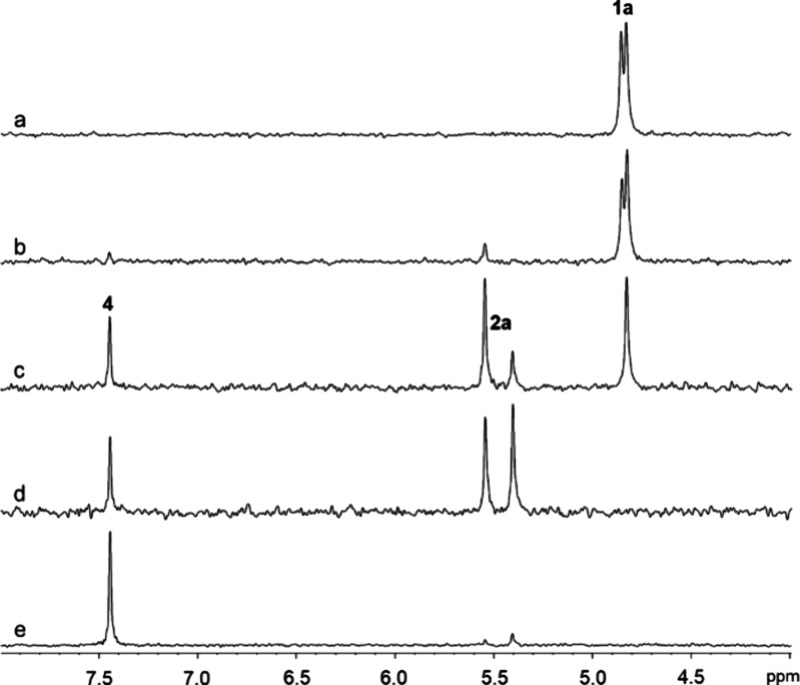
^31^P NMR spectra of compound **1a** at pH 8.0 and 30 °C. (a) Compound **1a** prior to the addition of carboxypeptidase Y; (b) partial hydrolysis of compound **1a** using 25 nM carboxypeptidase Y after 6 min; (c) partial hydrolysis of compound **1a** using 200 nM carboxypeptidase Y after 6 min; (d) complete hydrolysis of **1a** by 3.9 μM carboxypeptidase Y after 6 min; (e) final reaction product (**4**) formed with 200 nM carboxypeptidase Y after 30 min.

**Figure 5:**
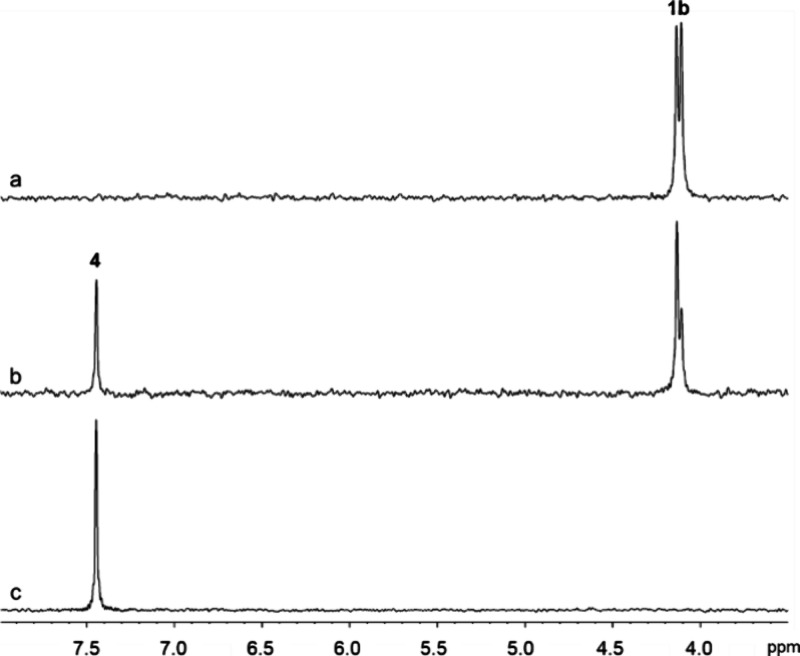
^31^P NMR spectra of compound **1b** at pH 8.0 and 30 °C. (a) Compound **1b** prior to the addition of carboxypeptidase Y; (b) partial hydrolysis of compound **1b** by 25 nM carboxypeptidase Y after 10 min; (c) complete hydrolysis of compound **1b** by 1.9 μM carboxypeptidase Y after 1 h.

Two pairs of diastereomeric ProTide analogs were synthesized as probes of the chemical reaction mechanism for prodrug activation. Both pairs of diastereomers contain an ethoxy substituent to mimic the modified nucleoside, and an l-alanyl isopropyl ester connected to the phosphorus core via an amide linkage. Compound **1a** contains a phenolic leaving group, whereas compound **1b** is substituted with *p*-nitrophenol (Figure 3). The *p*-nitrophenol substitution was made in an attempt to capture the putative cyclic intermediate by changing the rate of the spontaneous cyclization step (see Figure 2). The ^31^P NMR spectrum of compound **1a** is shown in Figure 4a. A single resonance is observed at ∼4.8 ppm for each diastereomer with a difference in chemical shift of ∼0.04 ppm. Similar results were obtained with compound **1b**. The ^31^P NMR spectrum of compound **1b** has a pair of resonances at ∼4.1 ppm with a chemical shift difference of ∼0.03 ppm (Figure 5a).

### Postulated Activation Mechanism

**Figure 6:**
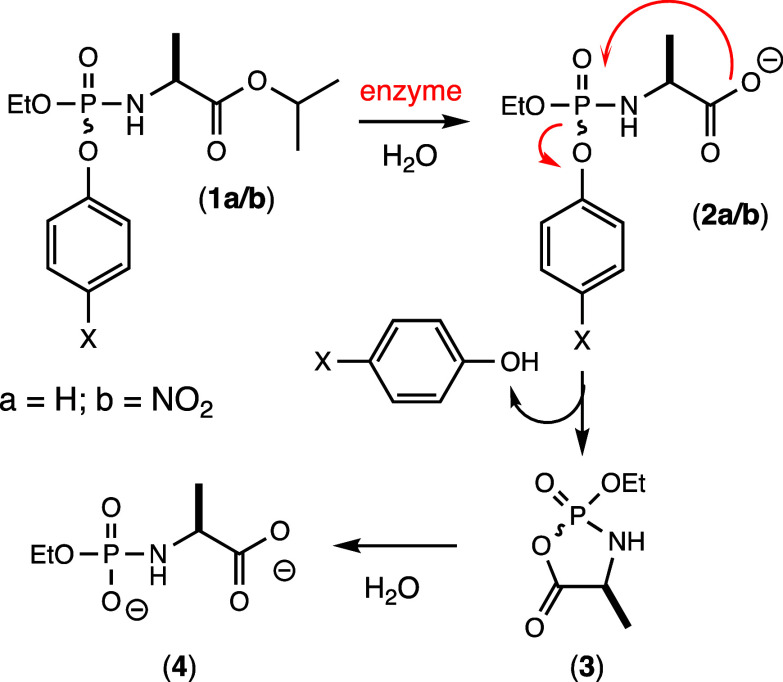
Working model for the activation of representative ProTides where X = H for **1a**, and X = NO_2_ for **1b**.^1,6^

The proposed activation mechanism for the ProTide analogues synthesized for this investigation is presented in Figure 6.^1,6,7,9,10,12,14,15,24^ In the first step the isopropyl substituent of compound **1** is hydrolyzed by carboxypeptidase Y to form compound **2**. The newly formed carboxylate spontaneously attacks the phosphorus center with expulsion of the phenol and formation of the putative cyclic intermediate **3**.^6,12^ In the final step, water attacks the mixed anhydride intermediate at either the phosphate or carboxylate center with the ultimate formation of compound **4**.^6,10^ For the enzyme-initiated transformation of **1** to **4**, support for the postulated cyclic intermediate **3** has been reported, although the site of attack by water has not been determined for the formation of **4** from intermediate **3**.^6,10,15,16^

### Hydrolysis of Compounds **1a** and **1b** by Carboxypeptidase Y

**Figure 7:**
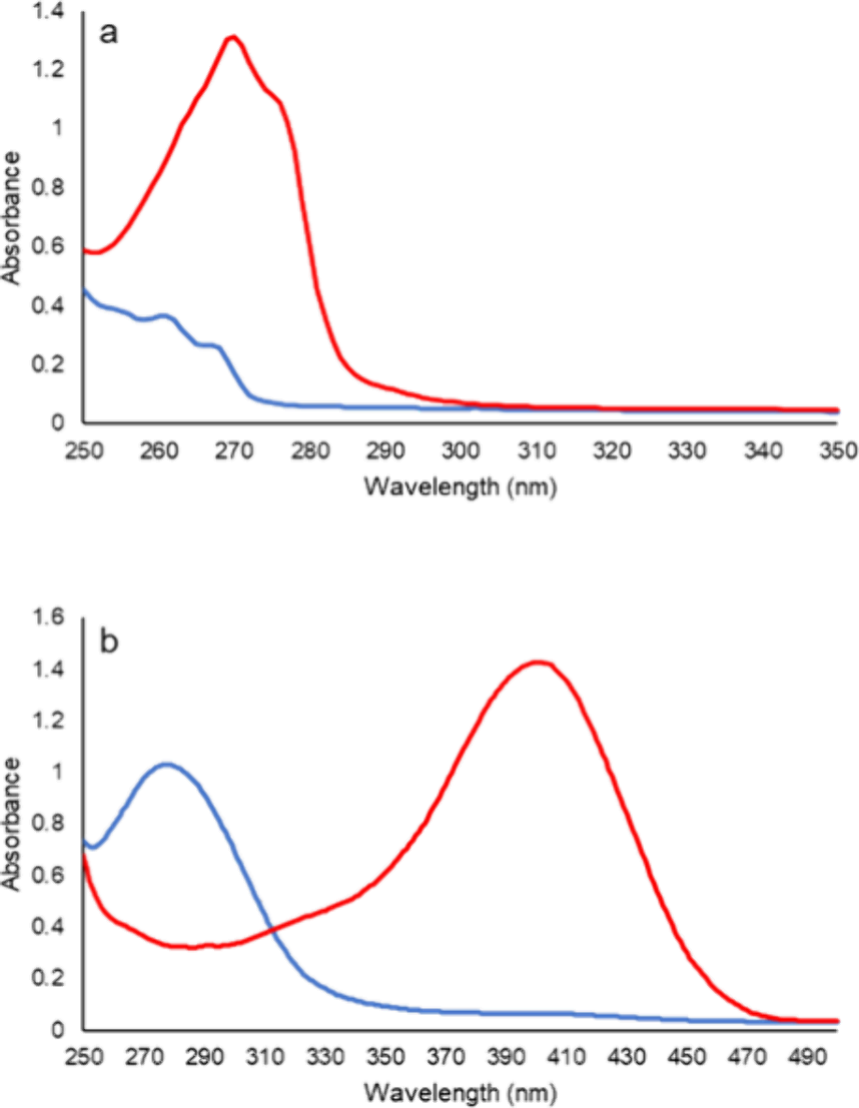
UV–vis spectra of compounds **1a** (1.0 mM) and **1b** (100 μM) before and after hydrolysis using 1.0 μM carboxypeptidase Y at pH 8.0 and 25 °C. (a) Compound **1a** prior to the addition of carboxypeptidase (blue); compound **1a** after the addition of carboxypeptidase Y (red). (b) compound **1b** prior to the addition of carboxypeptidase Y (blue); compound **1b** after the addition of carboxypeptidase (red).

We utilized carboxypeptidase Y to initiate the hydrolysis of the isopropyl ester from compounds **1a/b** and measured the release of either phenol or *p*-nitrophenol by monitoring the changes in the UV–vis spectrum as a function of time. The UV–vis spectrum of compound **1a** is shown in Figure 7a, and that of compound **1b** is in Figure 7b. After the addition of carboxypeptidase Y to compound **1a**, there is a significant increase in the absorbance at 271 nm due to the ultimate formation of phenol (red spectrum in Figure 7a) at pH 8.0 (Δε_271_ = 1,320 M^–1^ cm^–1^). For compound **1b**, there is a significant increase in the absorbance at 400 nm after the addition of carboxypeptidase Y due to the formation of *p*-nitrophenolate. However, the isosbestic point for *p*-nitrophenol is at 348 nm; therefore, reactions were monitored at this wavelength to avoid any variations due to pH (Δε_348_ = 4,840 M^–1^ cm^–1^).

### Stereoselectivity of Carboxypeptidase Y for Hydrolysis of Compounds **1a** and **1b**

The stereoselective preference for the hydrolysis of the carboxylate ester in compounds **1a** and **1b** by carboxypeptidase Y was initially interrogated using ^31^P NMR spectroscopy. After the addition of a small amount of carboxypeptidase Y (25 nM) to 4.0 mM of compound **1a**, one of the two diastereomers is clearly hydrolyzed faster than the other (Figure 4b) with formation of intermediate **2a**, depicted by the appearance of a new resonance at 5.54 ppm. Using a higher concentration of carboxypeptidase Y (200 nM), both diastereomers of intermediate **2a** (new resonance at ∼5.40 ppm) are observed in Figure 4c, along with the complete hydrolysis of the faster diastereomer of compound **1a**. The addition of a very high concentration of carboxypeptidase Y (3.9 μM) resulted in the rapid hydrolysis of both diastereomers of compound **1a** and formation of nearly equal amounts of the two diastereomers of intermediate **2a**, along with the ultimate formation of compound **4** (at 7.45 ppm) as shown in Figure 4d,e. No ^31^P resonances were observed for the putative cyclic intermediate **3**.

Carboxypeptidase Y also shows a stereoselective preference for the hydrolysis of compound **1b** (Figure 5b) where the most upfield resonance at ∼4.10 ppm is diminished relative to the resonance for the other diastereomer. In this case, no new resonances are observed for either of the two diastereomeric intermediates (**2b**), indicating that the cyclization of intermediate **2b** and expulsion of the *p*-nitrophenolate substituent is significantly faster than for the phenolate in intermediate **2a**. However, the same ultimate product (**4**) is observed at 7.45 ppm in Figure 5b,c. The relative rates of ester hydrolysis of compounds **1a** and **1b** by carboxypeptidase Y were determined by monitoring the changes in the ^31^P NMR spectra as a function of time after the addition of the enzyme to an equal mixture of compounds **1a** and **1b**. The time course is presented in Figure S1, and the relative rate of hydrolysis of the two compounds is ∼3.0. It is assumed here that the relative stereochemistry of the faster diastereomer is the same for compounds **1a** and **1b**, but the absolute stereochemistry of this isomer is unknown at this time.

### Rate of Cyclization Determined by ^31^P NMR Spectroscopy

The rate of cyclization of intermediate **2a** was measured by following the formation of compound **4** and the disappearance of **2a** by ^31^P NMR spectroscopy after a large excess of carboxypeptidase Y was added to compound **1a** (Figure 4d and Figure S2) and fitting the data to a single exponential using eq 1. The measured rate constant for the decay of compound **2a** and the formation of compound **4** is 0.096 ± 0.005 min^–1^, corresponding to a half-life of ∼7.2 min at 30 °C. A direct measurement for the cyclization of compound **2b** could not be obtained due to the very rapid rate of *p*-nitrophenol release after hydrolysis of the carboxylate ester by carboxypeptidase Y (reaction is complete in <10 s) with an estimated rate constant of ≥10 min^–1^. Therefore, **2b** cyclizes ≥100-fold faster than **2a**.

### Mass Spectrometry Analysis

**Figure 8:**
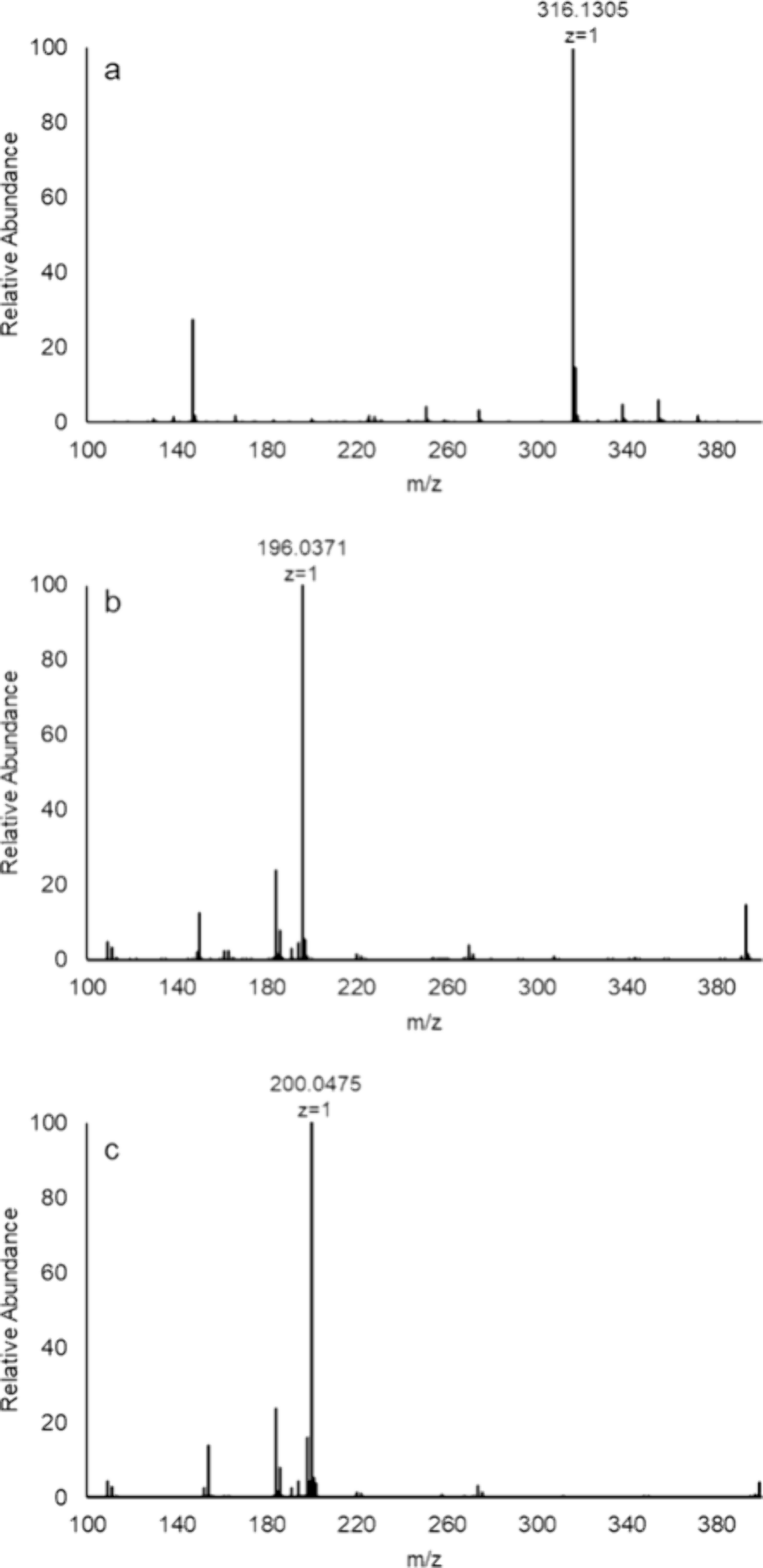
Mass spectra for compound **1a** and its hydrolysis products. (a) Positive ion ESI-MS of compound **1a** ([M+H]^+^ = 316.1305) prior to the addition of carboxypeptidase Y. (b) Negative ion ESI-MS for compound **4** ([M-H]^−^ = 196.0371) after initiation of the hydrolysis of compound **1a** with carboxypeptidase Y. (c) Negative ion ESI-MS for compound **4** ([M-H]^−^ = 200.0475) after initiation of the hydrolysis of compound **1a** with carboxypeptidase Y in ^18^O-labeled water.

Electrospray ionization mass spectrometry (ESI-MS) was conducted in the positive ion mode for the initial identification of **1a**, with an observed *m*/*z* for the [M+H]^+^ cation at 316.1305 Da (Figure 8a). After the entire reaction sequence was complete, compound **4** was identified in the negative ion mode for the [M-H]^−^ anion at 196.0371 Da (Figure 8b). When the reaction sequence was conducted in 90% ^18^O-labeled water, the final product was identified by the [M-H]^−^ anion at an *m*/*z* of 200.0475 Da, indicating the incorporation of two oxygen-18 atoms from two water molecules in the conversion of **1a** to **4** (Figure 8c).

**Figure 9:**
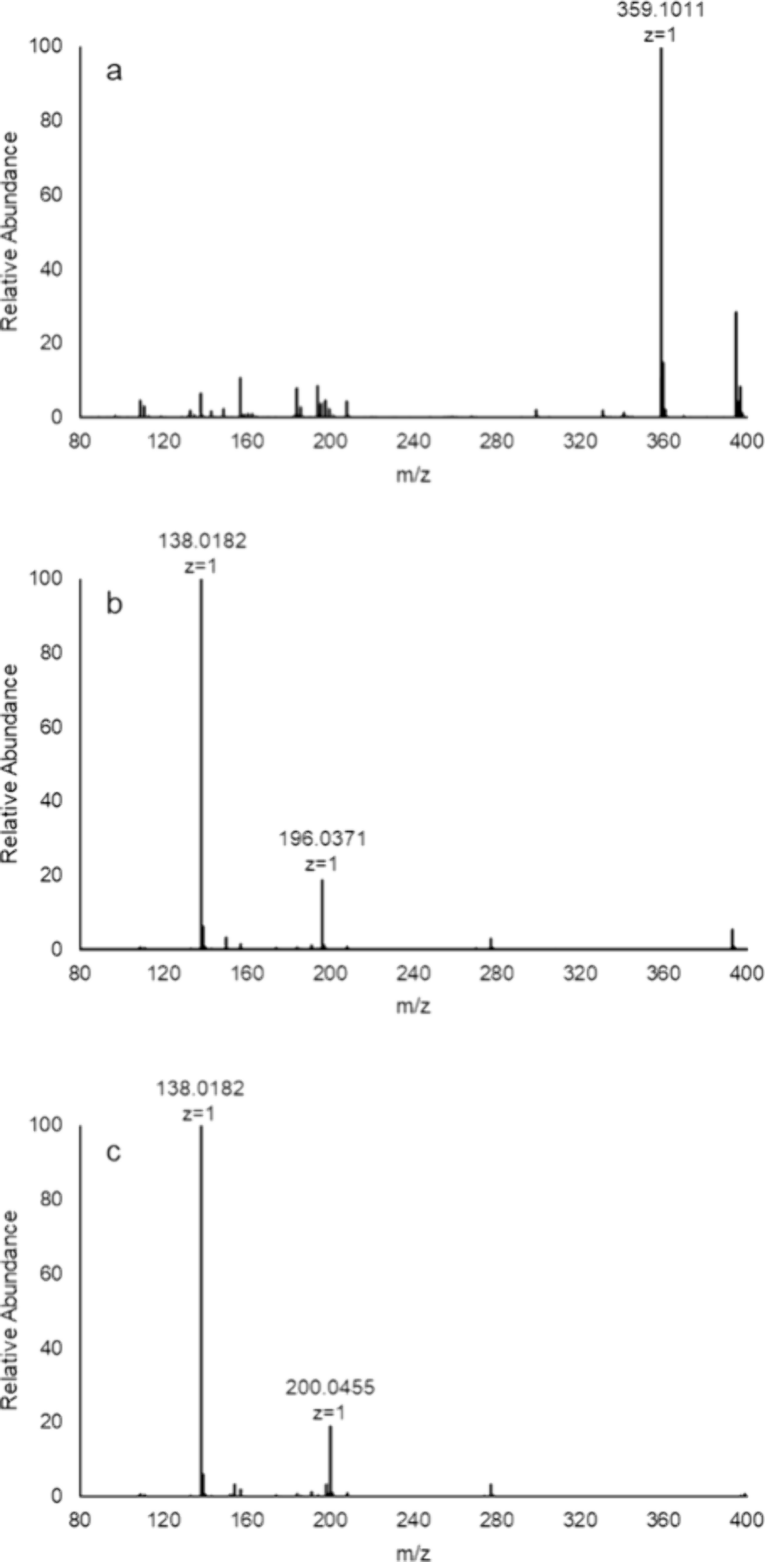
Mass spectra for compound **1b** and its hydrolysis products. (a) Negative ion ESI-MS of compound **1b** ([M-H]^−^ = 359.1011) prior to the addition of carboxypeptidase Y. (b) Negative ion ESI-MS for compound **4** ([M-H]^−^ = 196.0371) after initiation of the hydrolysis of compound **1b** with carboxypeptidase Y. (c) Negative ion ESI-MS for compound **4** ([M-H]^−^ = 200.0455) after initiation of the hydrolysis of compound **1b** with carboxypeptidase Y in ^18^O-labeled water. The hydrolysis product *p*-nitrophenol appears at an *m*/*z* of 138.0182 for the [M-H] anion in panels (b) and (c).

The reaction products for the activation of compound **1b** were also analyzed by ESI-MS. Compound **1b** was first identified by the [M-H]^−^ anion at an observed *m*/*z* of 359.1011 Da (Figure 9a). After the addition of carboxypeptidase Y, the reaction products were identified in the negative ion mode at an *m*/*z* of 138.0182 (*p*-nitrophenolate) and compound **4** at 196.0371 Da (Figure 9b). The final reaction product, produced using ^18^O-labeled water, is shown in Figure 9c with an *m*/*z* of 200.0455 Da, indicating the incorporation of two oxygen-18 atoms from the reactions involving two separate water molecules.

### Hydrolysis of Compounds **1a** and **1b** Monitored by UV–vis Spectroscopy

**Figure 10:**
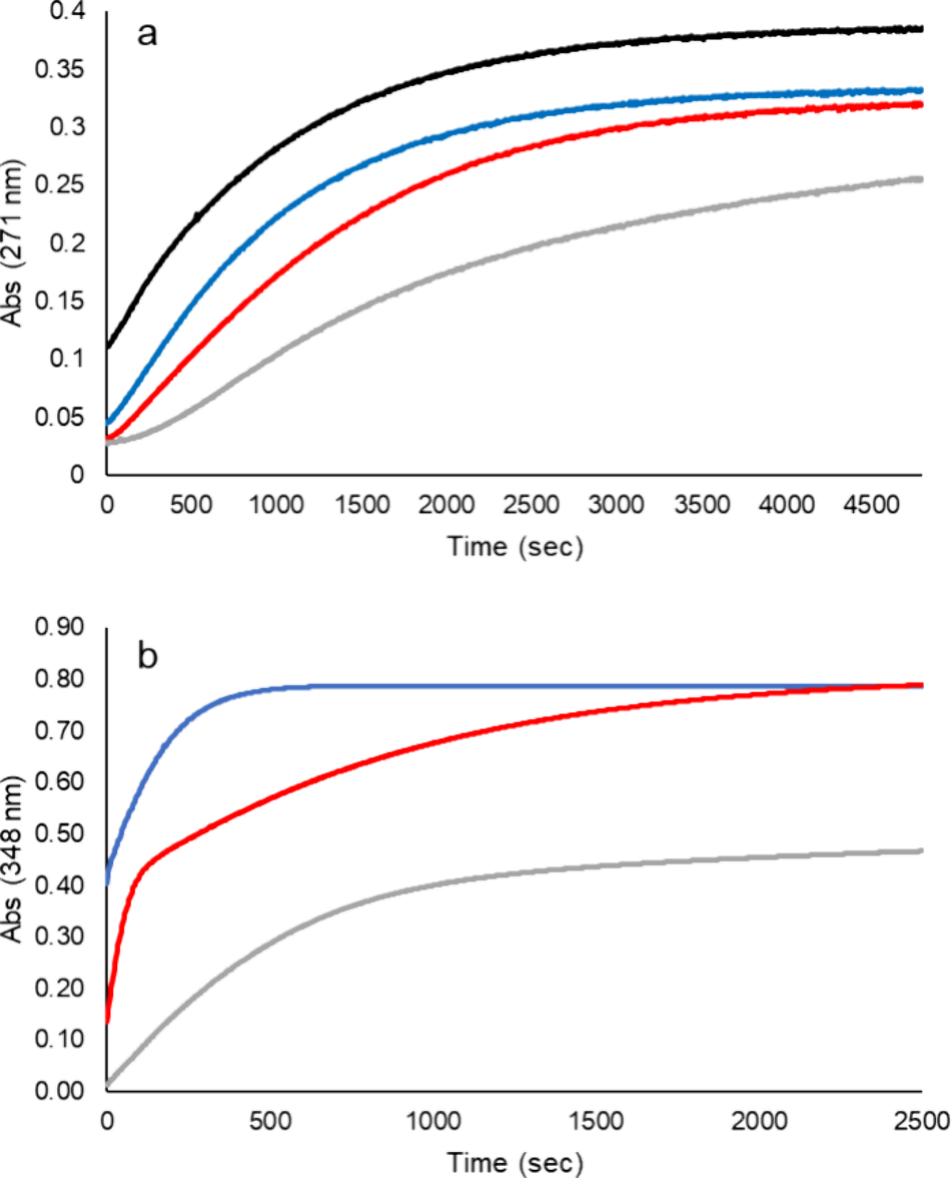
Time courses for the hydrolysis of compounds **1a** (400 μM) and **1b** (200 μM) by carboxypeptidase Y at pH 8.0 and 25 °C. (a) Time course for the hydrolysis of compound **1a** by increasing amounts of carboxypeptidase Y; 1.0 μM (black), 200 nM (blue), 50 nM (red), and 10 nM (gray). (b) Time courses for the hydrolysis of compound **1b** by carboxypeptidase Y; 100 nM (blue), 25 nM (red), and 5 nM.

To further probe the reaction mechanism for activation of compound **1a** by carboxypeptidase Y, the time course for the formation of phenol was monitored by UV–vis spectroscopy at 271 nm. Shown in Figure 10a are the time courses when different amounts of carboxypeptidase Y are added to compound **1a**. At low enzyme concentrations there is a definite lag phase that reflects the differential rate of hydrolysis of the two diastereomers of **1a** to **2a**, and the relatively slow rate of cyclization of intermediate **2a** to **4** via compound **3**. At the highest concentration of carboxypeptidase Y, the time course can be depicted as following a single exponential with a rate constant of 0.058 ± 0.001 min^–1^ for the conversion of **2a** to **3**/**4**. This rate constant is in reasonable agreement to the value of 0.096 min^–1^ determined by ^31^P NMR spectroscopy at a slightly higher temperature.

The time courses for the hydrolysis of compound **1b** by carboxypeptidase Y are exhibited in Figure 10b. Since the rate constant for the cyclization of **2b** to **3**/**4** is so much faster than for **2a** to **3**/**4** these assays provide a continuous monitor for the differential rate of hydrolysis of the two diastereomers of **1b** by carboxypeptidase Y. At the highest concentration of carboxypeptidase Y, the first phase is over within the dead-time of the assay, and at the lowest concentration of carboxypeptidase Y only a single phase is observed. However, at a concentration of 25 nM enzyme two approximately equal phases are observed and each phase could be fit to a single exponential with values of 1.39 ± 0.01 and 0.068 ± 0.001 min^–1^, thus indicating that the faster diastereomer is hydrolyzed ∼20 times faster than the slower diastereomer (Figure S3).

### Positional Hydrolysis of the Cyclic Intermediate

**Figure 11:**
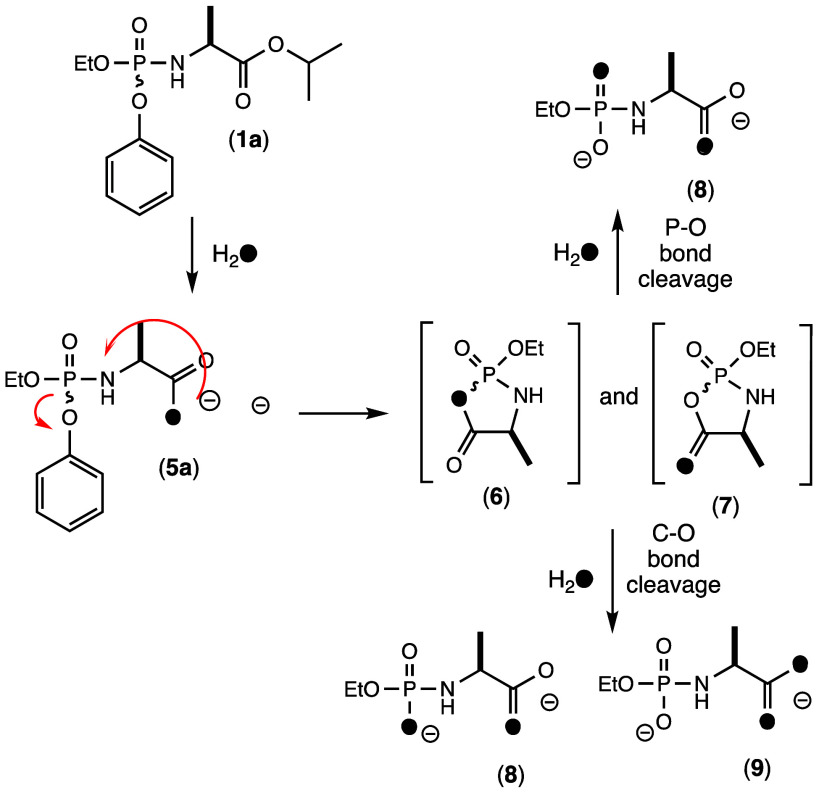
Possible reaction products for the activation of compound **1a** in ^18^O–H_2_O.

The hydrolysis of the putative cyclic intermediate **3** in the activation pathway shown in Figure 6 has two possible routes to product formation via either P–O or C–O bond cleavage. To determine the regiochemistry for product formation, the hydrolysis reactions were conducted using ^18^O-labeled water and the possible product outcomes are illustrated in Figure 11 using compound **1a** as an example. The initial hydrolysis of the isopropyl ester by carboxypeptidase Y in ^18^O-labeled water will result in the incorporation of a single ^18^O in the carboxylate product (**5a**). Cyclization of **5a** with the expulsion of phenol will result in an equal mixture of **6** and **7** where the ^18^O is distributed in the bridging (**6**) and nonbridging positions (**7**). If the attack of water occurs at the phosphorus center of the cyclic intermediate, then a single product (**8**) will be formed with one ^18^O-label attached to the phosphorus and the other attached to the carboxylate. Alternatively, if the attack of water occurs at the carboxylate center of the cyclic intermediate, then hydrolysis of **6** will produce **8**, but hydrolysis of **7** will produce **9**, where both ^18^O-labels are found within the carboxylate moiety. ESI-MS demonstrated for the activation of both **1a** and **1b** in ^18^O-labeled water that two ^18^O atoms are incorporated in the final product (Figures 8c and 9c) but did not differentiate the specific site of ^18^O-incorporation.

**Figure 12:**
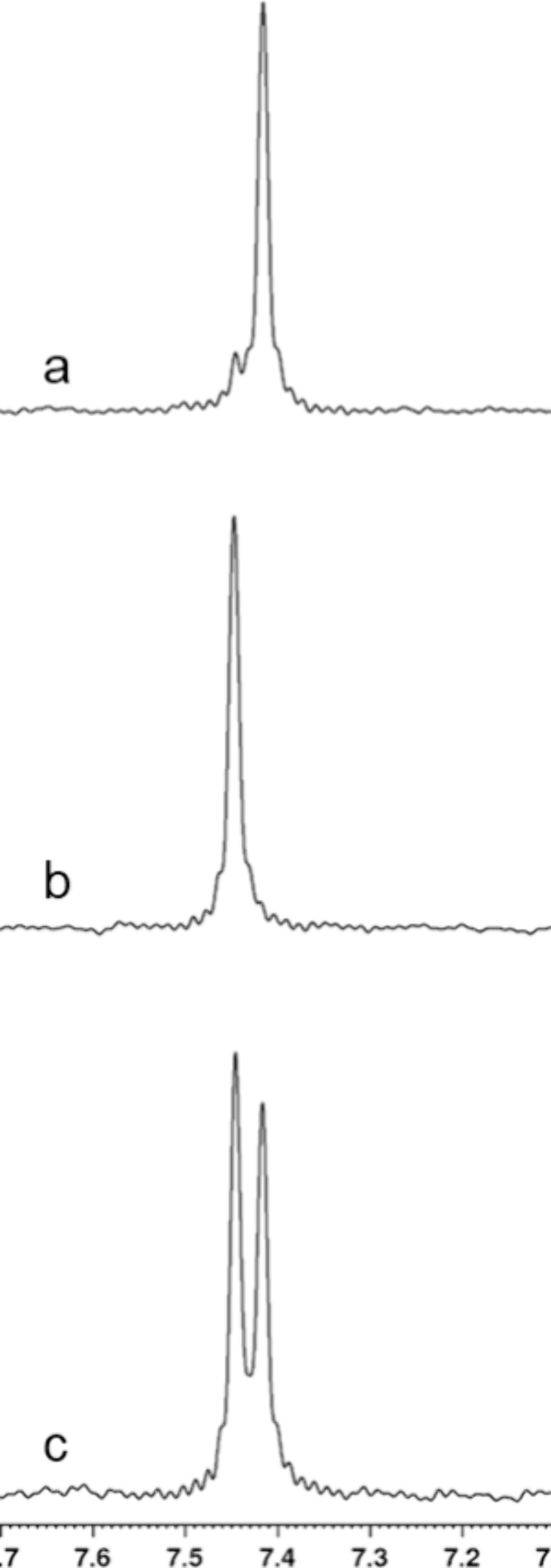
^31^P NMR spectra of the final reaction product when compound **1a** is activated by carboxypeptidase Y. (a) Final product when the reaction was conducted in ^18^O-water. (b) Final product when the reaction was conducted in ^16^O-labeled water. (c) Mixture of the products shown in spectra a and b.

The product outcome for the activation of compound **1a** was determined using ^31^P NMR spectroscopy. It has been demonstrated previously that the substitution of an ^18^O-isotope in phosphate esters will result in an upfield chemical shift difference of approximately 0.02–0.03 ppm.^27,28^ Therefore, if a mixture of **8** and **9** were to be formed via the hydrolysis of the carboxylate ester, the ^31^P NMR spectrum of the products would show two phosphorus resonances of equal intensity with a difference in chemical shift of ∼0.025 ppm. Alternatively, if **8** was exclusively formed via the attack of water on the phosphorus center then a single ^31^P NMR resonance would be observed. The ^31^P NMR spectrum of the final product when **1a** is activated in ^18^O-water is shown in Figure 12a. A single resonance is observed, demonstrating that water exclusively attacks the phosphorus center of the cyclic intermediate rather than the carboxylate center. To prove that the phosphate ester of the final product contains a single ^18^O-label, the same reaction was conducted in unlabeled water (Figure 12b) and then the two products were combined in equal ratios. The ^31^P NMR spectrum of the reaction mixture now shows two resonances separated by ∼0.03 ppm (Figure 12c). Again, this result demonstrates the attack at the phosphorus center in the putative cyclic intermediate formed during the activation of the ProTide analogue. An identical experiment was conducted using compound **1b** and the overall conclusion is the same (the ^31^P NMR spectra are presented in Figure S4).

## Conclusions

ProTide pharmaceuticals have become an effective method for the treatment of viral infections. These compounds have a modified nucleotide core where the phosphate moiety is further esterified with phenol and amidated with a derivatized l-alanine. The design of these prodrugs requires *in vivo* modification to facilitate subsequent cellular phosphorylation. In the proposed mechanism of activation, a cellular protease/esterase hydrolyzes a carboxylate ester from the l-alanyl moiety of the prodrug that subsequently cyclizes with the expulsion of the phenol. The putative cyclic intermediate then reacts with water to generate the phosphoramidate product. The nucleophilic attack of the cyclic intermediate could occur at either the phosphate or carboxylate center of the mixed anhydride intermediate. Here, we demonstrated that attack by water on the putative cyclic intermediate formed during the activation of ProTide analogues occurs exclusively at the phosphorus center. We also demonstrated that formation of the putative cyclic intermediate occurs more than 2 orders of magnitude faster with a *p*-nitrophenol derivative prodrug relative to one formed from phenol.

## Supporting Information Available

Additional ^31^P NMR spectra for the reactions of compounds **1a** and **1b** and the time course for hydrolysis of compound **1a** by carboxypeptidase Y (PDF)

## Accession Codes

Carboxypeptidase Y from *S. cerevisiae* (UniProt entry: P00729).
