# Supplementary material for: Regiochemical Analysis of the ProTide Activation Mechanism
Source: Biochemistry. 2024 Jul 3;63(14):1774–82. doi: 10.1021/acs.biochem.4c00176 (PMC11256751; doi:10.1021/acs.biochem.4c00176)
Supplement: Supplementary file 1 — bi4c00176_si_001.pdf [file bi4c00176_si_001.pdf]

SUPPORTING INFORMATION

Regiochemical Analysis of the ProTide Activation Mechanism

Kyle Glockzin[§], Tamari Narindoshvili[‡], and Frank M. Raushel[‡,§] *

[‡]Department of Chemistry, Texas A&M University, College Station, Texas US 77843

[§]Department of Biochemistry & Biophysics, Texas A&M University, College Station, Texas, US 77843

*To whom correspondence may be addressed: raushel@tamu.edu

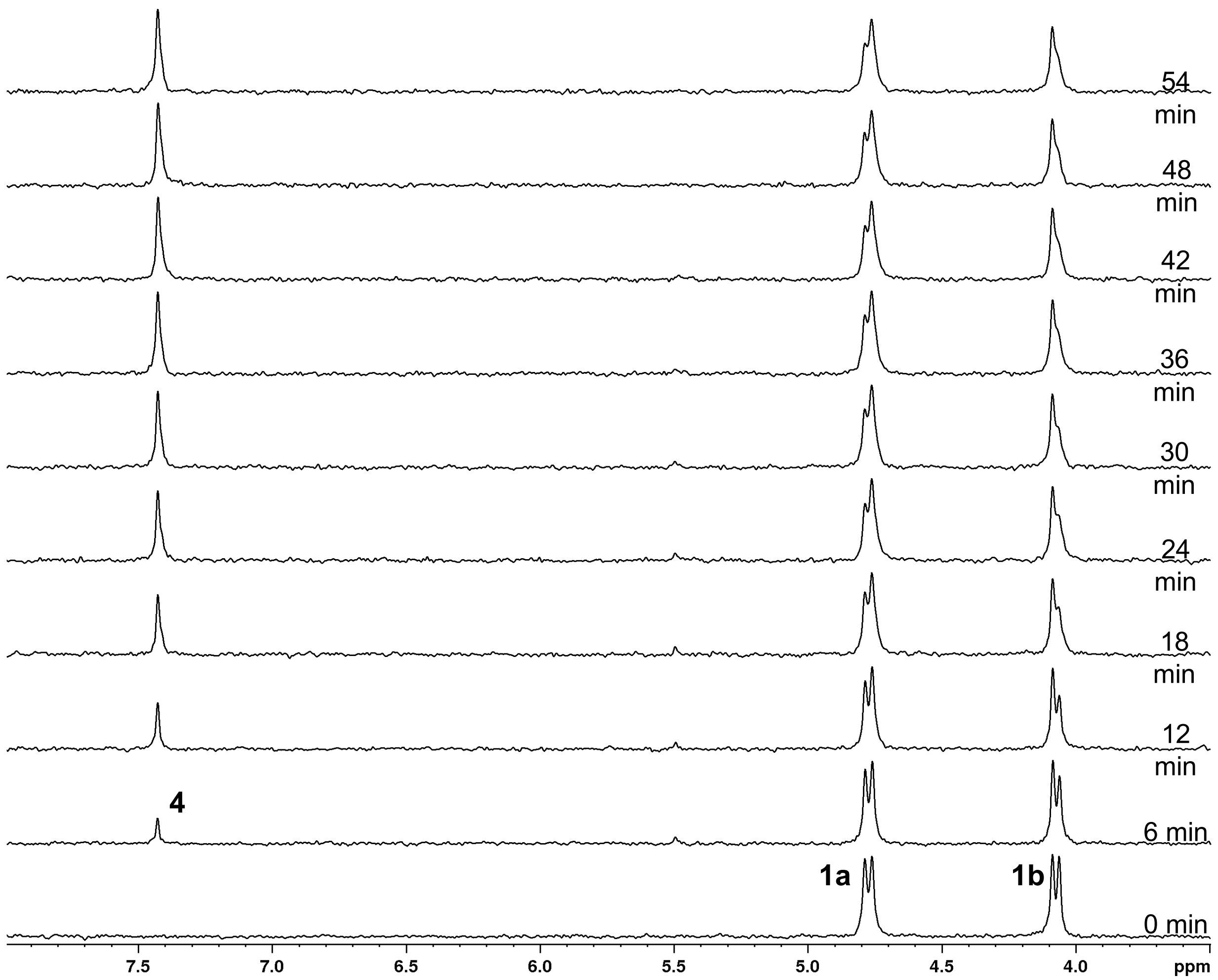

Figure S1: ^{31}P NMR spectra for the time course reaction of carboxypeptidase Y with an equal mixture of compounds **1a** and **1b**. Assay conditions were as follows; 100 mM triethanolamine-HCl (pH 8.0), 10% D_2O, 4.0 mM each of **1a** and **1b** (4% DMF), 30 °C, and 25 nM carboxypeptidase Y. Spectra were taken approximately every 6 min.

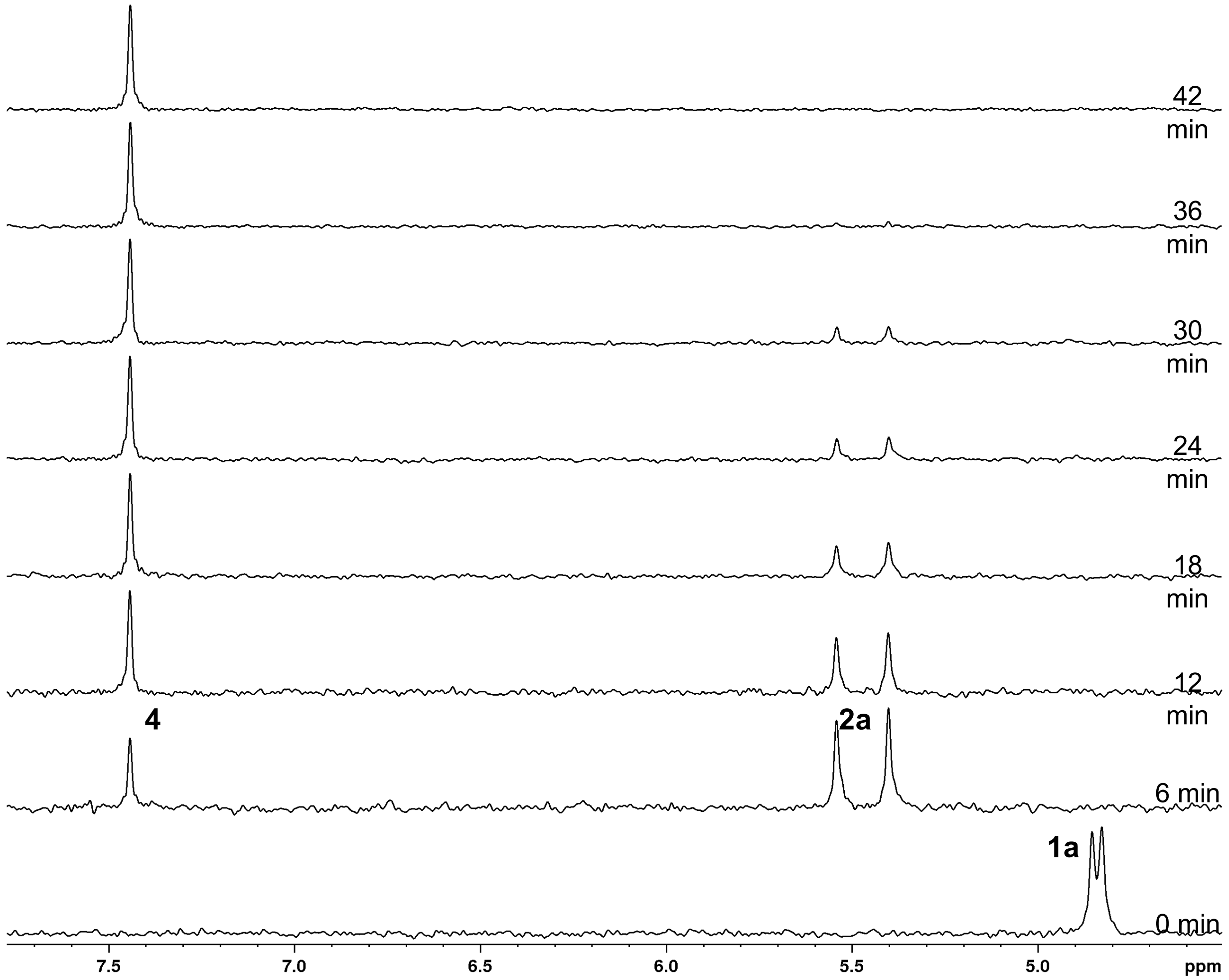

Figure S2: ^{31}P NMR demonstrating the decay of **2a** and subsequent formation of **4** after enzymatic hydrolysis of **1a**. Assay conditions were as follows; 100 mM triethanolamine-HCl pH 8.0, 10% D_2O, 4.0 mM **1a** (2% DMF), 30 °C , and 3.9 µM carboxypeptidase Y. Spectra were taken approximately every 6 min.

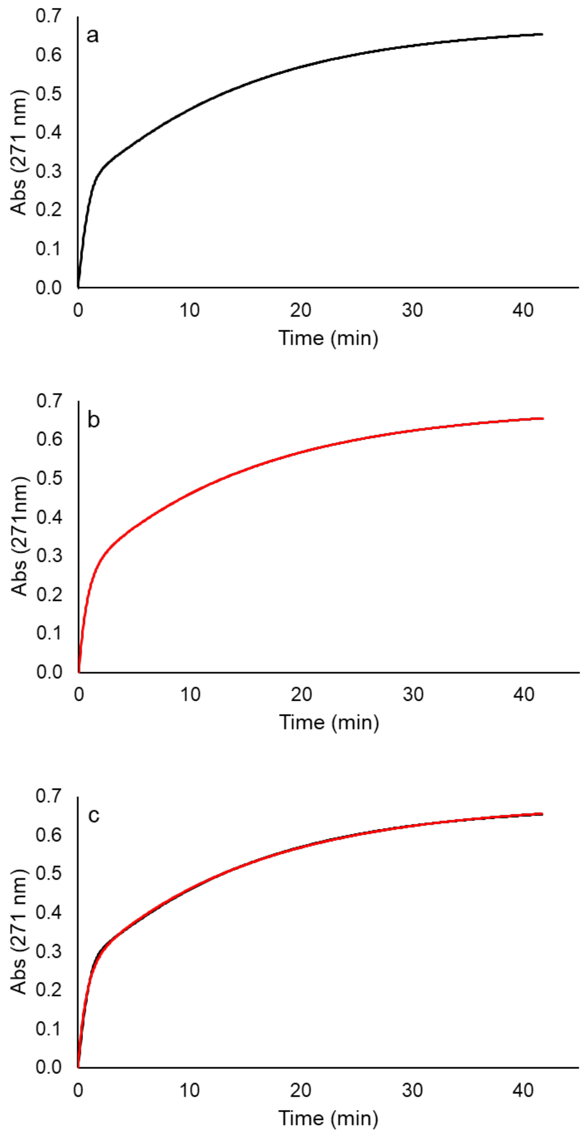

Figure S3: Time course for the hydrolysis of 200 μM compound **1b** (200 mM) by 25 nM carboxypeptidase Y at pH 8.0 (a) The change in absorbance at 348 nm monitoring the formation of *p*-nitrophenol as a function of time. (b) A fit of the data from panel a to equation 3. (c) a overlay of the time course from panel a with the fit of the data in panel b.

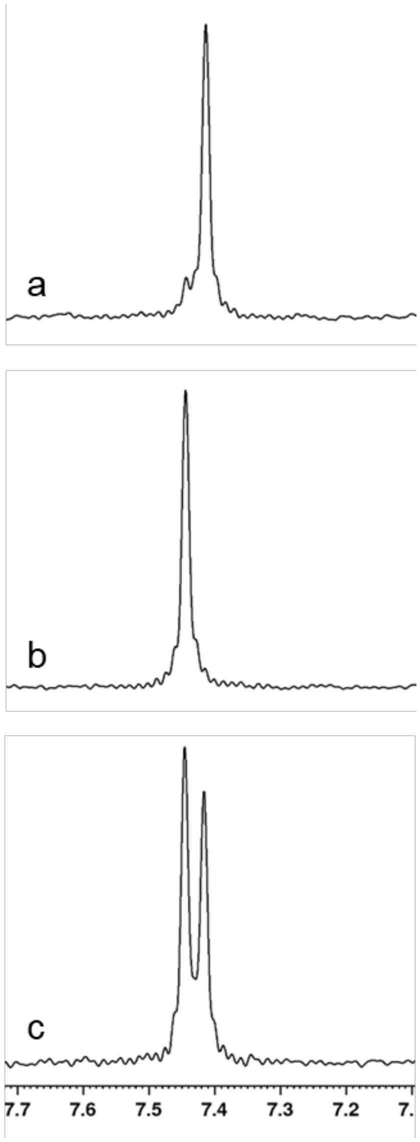

Figure S4: ^{31}P NMR spectra of compound **1b** (4.0 mM) hydrolyzed by 1.9 μM carboxypeptidase Y at pH 8.0 and 25 °C after 1 h. (a) – reaction products conducted in ^{18}O-water. (b) – reaction products conducted in ^{16}O-water. (c) – mixture of the ^{16}O- and ^{18}O-labeled reaction products (final products of a and b combined). The two resonances are separated by ~0.03 ppm.
